# The dual mediating effect of physical exercise on job performance: a conservation of resources perspective

**DOI:** 10.3389/fpsyg.2026.1780066

**Published:** 2026-03-06

**Authors:** Yuchun Yang, Mengsha Yuan, Hao Zhou, Xinshu Wu, Huizhu Xu

**Affiliations:** School of Economics and Management, Chengdu Sport University, Chengdu, China

**Keywords:** conservation of resources theory, exercise behavior, positive affect, self-efficacy, work outcomes

## Abstract

**Introduction:**

Physical exercise has been widely shown to benefit employees’ psychological functioning; however, less is known about how these benefits are translated into work-related outcomes. Drawing on Conservation of Resources (COR) theory, this study examines whether and how physical exercise influences job performance through the mediating roles of positive affect and self-efficacy.

**Methods:**

A three-wave time-lagged survey was conducted among full-time employees in China. Physical exercise was measured at Time 1, positive affect and self-efficacy at Time 2, and job performance at Time 3. The hypothesized mediation model was tested using PROCESS Model 4 with 5,000 bootstrap samples.

**Results:**

The results indicate that physical exercise significantly predicts both positive affect and self-efficacy, and that these psychological resources are positively associated with job performance. When positive affect and self-efficacy are included simultaneously in the model, the direct effect of physical exercise on job performance is substantially reduced and becomes non-significant, whereas the indirect effects through both mediators remain significant, underscoring the dominant role of the mediated pathways.

**Discussion:**

These findings suggest that the effects of physical exercise on job performance are primarily transmitted through psychological resources rather than through a direct mechanism. From a theoretical perspective, this study demonstrates that physical exercise functions as an upstream resource investment behavior whose performance benefits are mainly realized through the accumulation and deployment of psychological resources, thereby extending COR theory to the non-work domain. From a practical perspective, the results imply that organizational initiatives aimed at enhancing job performance through physical exercise should prioritize interventions that effectively foster employees’ positive affect and self-efficacy, rather than merely encouraging participation in exercise activities.

## 1 Introduction

In recent years, intensified social competition and expanding role expectations have exposed employees to multiple and persistent sources of stress across both work and non-work domains. These accumulated demands are frequently translated into physical and psychological strain (Ganster and Rosen, 2013). As a result, an increasing number of employees have adopted physical exercise as a self-regulatory strategy to relieve stress, stabilize emotional states, and maintain overall well-being (Stults-Kolehmainen and Sinha, 2014). It is essential to clarify that physical exercise, physical activity, and sport participation are related but conceptually distinct constructs. Physical activity is a broad umbrella term referring to any bodily movement produced by skeletal muscles that results in energy expenditure, encompassing behaviors occurring across multiple life domains, such as work, transportation, household activities, and leisure (Bull et al., 2020). Owing to this breadth, physical activity is characterized by substantial heterogeneity in both behavioral forms and psychological meanings. By contrast, physical exercise constitutes a specific subcategory of physical activity that is planned, structured, repetitive, and goal-oriented, with the explicit purpose of improving or maintaining physical health and psychological functioning (Caspersen et al., 1985). Compared with incidental or sporadic physical activity, physical exercise exhibits a more stable and consistent association with key psychological resources, such as positive affect and self-efficacy. Sport participation, in turn, encompasses a diverse set of sport-related engagements, including organized competitive involvement, sport event spectating, and sport-related consumption, some of which involve limited or no substantive physical exertion (Downward and Rasciute, 2011; Eime et al., 2013). Accordingly, the present study focuses on physical exercise as the core construct, as regular and purposefully structured exercise represents a central pathway through which affective and cognitive resources are generated. This focus is theoretically aligned with the study’s central concern regarding the cross-domain transfer of psychological resources from non-work contexts to the work domain.

A substantial body of literature has examined the effects of physical exercise on physical and mental health outcomes (Rebar et al., 2015). Prior studies have demonstrated that well-structured exercise can alleviate depression and stress (Salmon, 2001), contribute to improvements in both physical and psychological functioning, and has gradually become an indispensable component of modern lifestyles (Miller et al., 2016). Other research has indicated that voluntarily engaging in exercise may stimulate dopaminergic activation, enhance positive affect, and strengthen individuals’ psychological resilience when coping with adverse experiences (Dishman et al., 2006). Positive affect, in turn, has been shown to be associated with more adaptive psychological, cognitive, and behavioral responses (Steptoe et al., 2008). Collectively, these findings suggest a close relationship between physical exercise and the emotional states experienced by employees. Beyond these affective outcomes, physical exercise may also influence individuals’ cognitive appraisals of their own capabilities. Successfully attaining exercise-related goals—such as improving endurance, increasing muscular strength, or managing body weight—can reinforce individuals’ sense of competence. Prior research has provided empirical evidence that physical activity contributes to the development of higher levels of self-efficacy (McAuley et al., 2003). Originally proposed by Bandura (1982), self-efficacy refers to individuals’ beliefs regarding their capacity to effectively perform specific tasks (Bandura, 1995). Extensive research has established self-efficacy as a key predictor of motivation, persistence, learning capacity, and performance outcomes (Fida et al., 2025).

In recent years, several high-quality systematic reviews and meta-analyses have provided robust empirical evidence for the association between physical exercise and work-related outcomes. For instance, the systematic review by Zhang S. et al. (2025) demonstrated that workplace health promotion interventions centered on physical activity can significantly enhance employee productivity. Similarly Marin-Farrona et al. (2023) confirmed that physical activity–based interventions are effective in improving performance-related work outcomes. Integrative evidence synthesized by Schaller et al. further emphasized the sustained benefits of physical exercise for work engagement and productivity. Despite this growing body of evidence (White et al., 2016), these studies share notable limitations. Specifically, many reviews have focused primarily on establishing direct associations between exercise and work outcomes, without unpacking the underlying mediating mechanisms through which such effects occur (Sonnentag and Fritz, 2015). Others have treated physical exercise as a background health behavior, rather than analytically isolating its unique affective and cognitive resource–generation pathways (Shi et al., 2022). Although extensive research has examined how leadership styles, work environments, and psychological resources relate to job performance, relatively few studies have directly investigated whether and how physical exercise influences job performance. In particular, there remains a lack of integrative research explicating the core mechanism through which psychological resources generated by physical exercise are transferred across domains—namely, from non-work contexts into the workplace—and subsequently translated into performance outcomes. Existing studies have either remained confined to the health domain when examining the psychological benefits of exercise (Teychenne et al., 2025; White et al., 2017), or have failed to explicitly model the synergistic roles and cross-domain deployment of positive affect and self-efficacy as complementary psychological resources (Hobfoll et al., 2018). Taken together, these limitations underscore the need for further research. Accordingly, further research is warranted to clarify whether and how personal resources acquired through physical exercise can be transferred into the work domain and ultimately contribute to improved job performance.

These deficiencies highlight the necessity of additional research to supplement existing inadequacies. According to Conservation of Resources (COR) theory, individuals with sufficient resources are more capable of obtaining new resources, and initial resource gains can foster further resource acquisition, thereby forming gain spirals (Hobfoll and Wells, 1998). Against this backdrop, this study focuses on positive affect and self-efficacy as key mediating variables, constructs a dual-mediation model, and empirically tests the mechanism through which physical exercise influences job performance. The findings are expected to enrich the research on the non-work factors affecting job performance and provide targeted practical implications for organizations to improve employee performance through physical exercise interventions.

## 2 Theory and hypotheses

### 2.1 Physical exercise and job performance

According to Conservation of Resources (COR) theory (Hobfoll, 1989), individuals are motivated to acquire, retain, and protect valuable resources. Resource loss causes significant stress, whereas resource gain enhances psychological resilience and promotes the accumulation of additional resources over time, forming a resource gain pathway. Employees with more resources are better able to resist resource loss, acquire new resources, and integrate them into a resource caravan, thereby reducing stress and burnout while enhancing work engagement and motivation (Hobfoll, 2011; Wheeler et al., 2012).

Physical exercise is primarily a behavior performed outside of the work environment, and, unlike job training or task practice, it does not directly modify job-specific skills, work strategies, or organizational processes. Instead, the benefits of physical exercise are realized through enhancements in psychological resources that can be transferred across life domains and subsequently applied in work contexts (Biddle et al., 2019; Xanthopoulou et al., 2007). As a proactive resource investment behavior, physical exercise initiates these resource gain pathways, enabling individuals to accumulate psychological resources that are transferable across domains. In particular, positive affect and self-efficacy generated through exercise form a complementary resource caravan, where affective and cognitive resources mutually reinforce each other: positive affect provides emotional energy that supports coping and resource mobilization, while self-efficacy enhances confidence and goal-directed effort, sustaining and amplifying positive affect. Through this synergistic interaction, these resources can be applied in the workplace to support employees in managing work demands and improving job performance (Chen et al., 2015; Halbesleben et al., 2014; Hobfoll et al., 2018).

Empirical evidence supports this COR-based perspective. Physical activity has been shown to relieve work-related stress and promote resource accumulation (Park and Jang, 2019), employees with higher fitness levels cope better with job demands and stressors (Gerber et al., 2013), and adherence to corporate fitness programs is positively associated with performance outcomes (Mills et al., 2007). Moreover, prior studies have confirmed that the physical and psychological benefits of exercise can transfer to the work domain, enhancing job performance–related indicators (Marin-Farrona et al., 2023; White et al., 2016; Zhang S. et al., 2025). This study maintains that physical exercise can improve job performance.

*Hypothesis 1*: Physical exercise is positively related to job performance.

### 2.2 The mediating role of positive affect

According to Conservation of Resources (COR) theory, resources are those objects, personal characteristics, conditions, or energies that are valued by the individual or that serve as a means for the attainment of other resources (Hobfoll, 2001). Positive affect represents a valued energy resource, capturing individuals’ subjective experiences of pleasure and well-being in favorable situations (Seligman, 2008). Physical activity has been shown to alleviate anxiety and improve mental health, facilitate the attainment of positive affect, and buffer against the detrimental impacts of negative emotional states (Greenwood, 2019). Engaging in physical exercise allows individuals to temporarily disengage from stressors, regulate emotions, and restore depleted emotional energy, thereby fostering more frequent and stable experiences of positive affect. In organizational contexts, employees who frequently experience positive affect tend to exhibit greater engagement and creativity, demonstrate stronger perseverance in challenging tasks, and display higher levels of motivation, which may ultimately enhance their job performance (Tsai, 2001; Wan et al., 2022; Watson et al., 1988). From the COR perspective, when individuals obtain positive affect as an energy resource, they tend to generate further resources, and such a positive cycle constitutes a gain spiral. Specifically, positive affect enhances flexibility, and supports sustained effort, which in turn facilitates the accumulation of additional psychological and motivational resources. Importantly, because physical exercise is typically performed in the non-work domain and does not directly alter job-specific skills or task strategies, its influence on work outcomes is more likely to occur through the accumulation of such transferable psychological resources rather than through direct performance-related pathways. This resource can be accumulated in non-work domains and subsequently deployed and utilized in the work domain. Accordingly, it is reasonable to hypothesize that positive affect may mediate the relationship between physical exercise and job performance.

*Hypothesis 2*: Positive emotion mediates the relationship between physical exercise and job performance.

### 2.3 The mediating role of self-efficacy

Self-efficacy represents another important psychological mechanism through which physical exercise may influence work outcomes. From the perspective of Conservation of Resources (COR) theory, self-efficacy constitutes a personal characteristics resource that reflects individuals’ beliefs in their capacity to successfully perform tasks and cope with challenges (Hobfoll, 1989, 2001). Such resources play a critical role in buffering stress and motivating further resource investment.

Although self-efficacy is often conceptualized as a relatively stable personal characteristic, prior research suggests that it can be strengthened through repeated mastery experiences and positive feedback within specific behavioral contexts. Physical exercise, as a goal-directed activity occurring in the non-work domain, provides individuals with opportunities for skill acquisition, goal attainment, and social interaction. Successfully completing exercise tasks, achieving fitness-related goals, and receiving social reinforcement during exercise participation can enhance individuals’ sense of competence and control, thereby promoting higher levels of self-efficacy (Mu et al., 2024). This process highlights the role of physical exercise in generating psychological resources outside the workplace.

Individuals with higher self-efficacy tend to set more challenging goals, persist in the face of obstacles, and recover more quickly from setbacks. Consistent with this theoretical reasoning, empirical studies have demonstrated that self-efficacy is positively associated with favorable job attitudes and higher levels of job performance (Carter et al., 2018; Stajkovic and Luthans, 1998). Self-efficacious employees are more likely to mobilize cognitive and behavioral resources to meet task demands and maintain effective functioning under pressure.

From a COR perspective, once self-efficacy is acquired as a valuable personal resource, individuals are motivated to protect, maintain, and invest this resource in their work roles. The investment of self-efficacy in the workplace facilitates sustained effort, effective problem solving, and adaptive goal-directed behavior, which ultimately contributes to improved job performance (Na-Nan et al., 2019). Importantly, this pathway suggests that physical exercise does not directly enhance job performance, but rather exerts its influence indirectly by fostering psychological resources that are transferable across life domains.

Based on this theoretical framework and prior empirical evidence, the present study proposes that self-efficacy mediates the relationship between physical exercise and job performance (Judge et al., 2007).

*Hypothesis 3*: Self-efficacy mediates the relationship between physical exercise and job performance.

## 3 Materials and methods

### 3.1 Participants

Data were collected from full-time employees working in Yunnan, Sichuan, and Shanxi Provinces in China. To reduce common method bias, a multi-wave research design was employed, with questionnaires administered at three separate time points with a one-month interval between each waves. This one-month time lag was adopted based on theoretical expectations of relatively rapid changes in affective states, self-efficacy, and performance-related processes (Dalal et al., 2020), and is consistent with methodological recommendations that time lags should correspond to the expected temporal dynamics of the focal constructs rather than being arbitrarily determined (Dormann and Griffin, 2015). At Time 1, 306 employees were invited to complete a survey assessing their participation in physical exercise, and 264 valid responses were obtained (response rate = 86.3%). At Time 2, the same participants reported their levels of positive affect and self-efficacy, yielding 253 valid responses (response rate = 82.7%). At Time 3, participants completed a self-report measure of job performance, resulting in 265 valid questionnaires (response rate = 86.7%). After matching responses across the three waves, a final sample of 238 complete cases was retained, corresponding to an effective response rate of 77.8%.

The final sample included employees with diverse marital statuses, ages, and educational backgrounds. Participation was entirely voluntary, and all respondents were informed that their responses would remain confidential and be used solely for academic research purposes. Attrition analyses comparing participants who remained in the final sample with those who dropped out revealed no significant differences in demographic characteristics or key study variables, indicating minimal attrition bias.

### 3.2 Measures

All scales were translated into Chinese following a translation–back translation procedure to ensure conceptual equivalence. Unless otherwise noted, all items were rated on a five-point Likert scale ranging from 1 = “strongly disagree” to 5 = “strongly agree.” Reliability analyses indicated that all measures demonstrated acceptable to excellent internal consistency in the present study.

#### 3.2.1 Physical exercise

Physical exercise was assessed using the Physical Activity Rating Scale (PARS-30) revised by Liang (1994). The total exercise volume score was calculated as intensity × time × (frequency - 1), with each dimension rated on five levels (each scored 1–5 points respectively). According to the scoring guidelines, ≤19 indicates a low level of exercise, 20–42 indicates a moderate level, and ≥43 indicates a high level of exercise. The Cronbach’s *α* for this scale in the present study was 0.722, indicating acceptable reliability. Importantly, the Chinese version of PARS-30 has been validated in prior research, demonstrating good test–retest reliability (ICC = 0.82) and applicability in Chinese samples for assessing physical activity behaviors (Zhang J. et al., 2025).

#### 3.2.2 Positive affect

Positive affect was measured using five items with the highest factor loadings from the Positive and Negative Affect Schedule (PANAS) developed by Watson et al. (1988). Sample items include “I feel happy,” “I feel enthusiastic,” and “I feel active.” The Cronbach’s *α* for this scale in the present study was 0.931, indicating excellent internal consistency. The Chinese version of PANAS, including short forms such as the I-PANAS-SF, has demonstrated satisfactory psychometric properties in Chinese populations, including a stable two-factor structure, strong internal consistency (*α* > 0.80), and measurement invariance across gender and age groups (Huang et al., 2003), supporting the reliability and validity of the affective measures used in this study.

#### 3.2.3 Self-efficacy

Self-efficacy was assessed using the Chinese version of the General Self-Efficacy Scale (GSES) developed by Schwarzer and Jerusalem (1995). The scale includes 10 items that assess individuals’ confidence in coping with difficulties and setbacks. Higher scores indicate higher levels of general self-efficacy. In the present study, the Cronbach’s *α* was 0.928, demonstrating excellent reliability.

#### 3.2.4 Job performance

Job performance was measured using the four-item scale developed by Chen et al. (2002). Employees rated their own task performance; a sample item is “I am able to complete my assigned job tasks on time.” The Cronbach’s α for this measure was 0.855, indicating good reliability.

### 3.3 Procedure

Data were collected using a three-wave time-lagged survey design with a one-month interval between each wave to reduce common method bias. At Time 1, employees reported their participation in physical exercise. At Time 2, the same respondents completed measures of positive affect and self-efficacy. At Time 3, they reported their job performance. After matching responses across the three waves, a total of 238 complete and valid cases were obtained for analysis.

Participation was entirely voluntary. The questionnaires were administered for research purposes only, and all responses were treated as confidential.

### 3.4 Statistical analysis

All statistical analyses were conducted using SPSS 26.0 and AMOS. First, the reliability of each scale was examined by Cronbach’s α coefficients to assess internal consistency. To further evaluate the construct validity of the study variables, confirmatory factor analysis (CFA) was performed in AMOS, and the fit indices of alternative models were compared.

Given that the data were collected through self-report questionnaires, Harman’s single-factor test was applied to assess the potential presence of common method bias. In addition, descriptive statistics and Pearson correlation analyses were conducted to obtain the means, standard deviations, and correlations among all study variables.

To test the hypothesized mediation model, the PROCESS macro (Model 4) was used in SPSS. Physical exercise was entered as the independent variable, positive affect and self-efficacy as mediating variables, and job performance as the dependent variable, with control variables included in the model. A bootstrapping procedure with 5,000 resamples and 95% confidence intervals was applied to estimate the indirect effects. Mediation effects were considered significant when the confidence intervals did not include zero.

## 4 Results

### 4.1 Descriptive analysis

Correlation analyses revealed that physical exercise was positively associated with positive affect (*r* = 0.162, *p* < 0.05) and self-efficacy (*r* = 0.214, *p* < 0.01). In addition, both positive affect (*r* = 0.343, *p* < 0.01) and self-efficacy (*r* = 0.355, *p* < 0.01) showed significant positive relationships with job performance. The means, standard deviations, and zero-order correlations among all study variables are summarized in Table 1, and together these results indicate consistent positive associations among the core constructs examined in this study.

**Table 1:** Means, standard deviations, and correlation coefficients of the study variables.

Variable	1	2	3	4	5	6	7	8
1 Gender								
2 Age	0.010							
3 Education level	0.050	0.038						
4 Marital status	−0.064	−0.605**	−0.066					
5 Physical exercise	−0.298**	0.075	0.086	0.170**				
6 Positive affect	−0.034	0.038	0.040	−0.024	0.162*			
7 Self-efficacy	−0.101	0.061	0.001	−0.083	0.214**	0.561**		
8 Job performance	−0.156*	0.229**	0.017	−0.247**	0.160*	0.343**	0.355**	
M	1.580	2.610	2.920	1.510	21.181	3.513	3.696	3.777
SD	0.495	1.573	0.786	0.557	20.499	0.781	0.558	0.630

*N* = 238, *p < 0.05, **p < 0.01, ***p < 0.001.

### 4.2 Reliability and validity analysis

Cronbach’s *α* coefficients indicated satisfactory to excellent internal consistency across all study measures, including physical exercise (*α* = 0.722), positive affect (α = 0.931), self-efficacy (α = 0.928), and job performance (*α* = 0.855).

Confirmatory factor analyses further supported the adequacy of the measurement model. The hypothesized four-factor model demonstrated a good fit to the data (*χ*^2^ = 494.157, df = 203, *χ*^2^/df = 2.434, RMSEA = 0.078, CFI = 0.914, TLI = 0.902, SRMR = 0.037), providing evidence for the discriminant validity of the focal constructs. In addition, results of Harman’s single-factor test showed that the largest factor accounted for 39.719% of the total variance, which falls below the recommended 40% threshold, suggesting that common method bias was unlikely to pose a serious threat in the present study. The model fit indices and common method bias diagnostics are reported in Table 2.

**Table 2:** Results of confirmatory factor analysis.

Model	Structure	*χ^2^*	*df*	*χ^2^/df*	RMSEA	CFI	TLI
Four-factor model	A;B;C;D	494.157	203	2.434	0.078	0.914	0.902
Three-factor model	A + B;C;D	640.173	206	3.108	0.094	0.872	0.856
Two-factor model	A + B + C;D	1274.064	208	6.125	0.147	0.685	0.650
One-factor model	A + B + C + D	1625.041	209	7.775	0.169	0.581	0.537

### 4.3 Hypothesis testing

Hierarchical regression analyses were conducted using SPSS 26.0 to test the proposed hypotheses, and the results are presented in Table 3. First, physical exercise exhibited a significant positive effect on job performance (Model M6, *β* = 0.158, *p* < 0.05), indicating that higher levels of physical exercise are associated with better job performance. Thus, Hypothesis 1 was supported.

**Table 3:** Hierarchical regression results.

Category	Positive Affect T2		Self-Efficacy T2		Job Performance T3						
	M1	M2	M3	M4	M5	M6	M7	M8	M9	M10	M11
Control variables T1											
Gender	−0.037	0.013	−0.107	−0.040	−0.170**	−0.124	−0.157**	−0.129*	−0.135*	−0.112	−0.120*
Age	0.036	−0.011	0.012	−0.051	0.118	0.075	0.106	0.079	0.114	0.091	0.087
Education level	0.041	0.021	0.001	−0.025	0.009	−0.008	−0.004	−0.015	0.009	−0.001	−0.008
Marital status	−0.001	−0.057	−0.082	−0.158	−0.186*	−0.237**	−0.186	−0.219*	−0.160*	−0.189*	−0.195**
Independent variable T1											
Physical exercise	—	0.174*	—	0.234***	—	0.158*	—	0.103	—	0.087	0.077
Mediating variable T2											
Positive affect	—	—	—	—	—	—	0.329***	0.314***	—	—	0.215**
Self-efficacy	—	—	—	—	—	—	—	—	0.322***	0.304***	0.185**
*F*	0.251	1.409	1.088	3.172**	6.441***	6.344***	12.141***	10.609***	11.680***	10.052***	10.289***
Δ*F*	0.251	6.019*	1.088	11.318***	6.441***	5.464*	31.564***	17.159***	29.487***	15.654***	9.491**
*R* ^2^	0.004	0.029	0.018	0.064	0.100	0.120	0.207	0.216	0.201	0.207	0.238
Δ*R*^2^	0.004	0.025*	0.018	0.046***	0.100***	0.021*	0.108***	0.116***	0.102***	0.107***	0.031**

*p < 0.05, **p < 0.01, ***p < 0.001.

Further analyses examining the effects of physical exercise on psychological resource variables showed that physical exercise significantly and positively predicted positive affect (Model M2, *β* = 0.174, *p* < 0.05) as well as self-efficacy (Model M4, β = 0.234, *p* < 0.001). These findings suggest that engaging in physical exercise contributes to enhanced positive emotional experiences and stronger perceptions of self-efficacy.

With respect to the mediating effects, the results indicated that positive affect was positively associated with job performance (Model M7, *β* = 0.329, *p* < 0.001). When both physical exercise and positive affect were simultaneously entered into the regression model (Model M8), positive affect remained a significant predictor of job performance (*β* = 0.314, *p* < 0.001), whereas the direct effect of physical exercise became non-significant (*β* = 0.103, *p* > 0.05). This pattern suggests that the effect of physical exercise on job performance is largely transmitted through positive affect, providing support for Hypothesis 2.

Similarly, self-efficacy showed a significant positive relationship with job performance (Model M9, *β* = 0.322, *p* < 0.001). When physical exercise and self-efficacy were included simultaneously in the model (Model M10), self-efficacy continued to exert a significant positive effect on job performance (*β* = 0.304, *p* < 0.001), while the direct effect of physical exercise was no longer significant (*β* = 0.087, *p* > 0.05). These results indicate that self-efficacy plays a dominant indirect role in linking physical exercise to job performance, thereby supporting Hypothesis 3.

Finally, when positive affect and self-efficacy were entered together into the regression model (Model M11), both positive affect (*β* = 0.215, *p* < 0.01) and self-efficacy (*β* = 0.185, *p* < 0.01) remained significant predictors of job performance, whereas the direct effect of physical exercise continued to be non-significant (*β* = 0.077, *p* > 0.05). Taken together, these findings further demonstrate that physical exercise influences job performance primarily through its indirect effects on key psychological resources, namely positive affect and self-efficacy.

This study further adopted Model 4 in the PROCESS macro to test the mediation effects with 5,000 bootstrap samples. Bootstrap estimates of the indirect effects, as reported in Table 4, further supported this interpretation. Specifically, the indirect effect through positive affect was significant (indirect effect = 0.030, SE = 0.018, 95% CI [0.002, 0.072]), and the indirect effect through self-efficacy was also significant (indirect effect = 0.044, SE = 0.030, 95% CI [0.003, 0.117]). Although the total effect of physical exercise on job performance remained significant (total effect = 0.146, SE = 0.059, 95% CI [0.030, 0.261]), the pattern of non-significant direct effect combined with significant indirect effects suggests that psychological mediators are the primary mechanisms linking exercise to job performance, rather than a direct effect of exercise itself. Thus, H2 and H3 were further supported.

**Table 4:** Bootstrap mediation analysis results.

Path	Effect	BootSE	BootLLCI	BootULCI
Total effect	0.146	0.059	0.030	0.261
positive affect	0.030	0.018	0.002	0.072
self-efficacy	0.044	0.030	0.003	0.117

These findings not only highlight the dominant role of the indirect pathways through positive affect and self-efficacy but also highlight the importance of clarifying how positive affect and self-efficacy are transformed into psychological resources to influence job performance. Based on Conservation of Resources (COR) Theory, this study further elaborates the internal psychological pathways of these mediators, focusing on the resource gain pathway and the resource caravan concept (Halbesleben et al., 2014).

From the perspective of COR Theory’s resource gain pathway, positive affect—induced by physical exercise—is a key psychological resource that can be accumulated to generate additional resources and promote job performance (Xanthopoulou et al., 2007). Specifically, positive affect enhances employees’work engagement, reduces stress-induced burnout, and facilitates the accumulation of complementary work resources (e.g., attention, interpersonal harmony) (Bakker and Demerouti, 2017; Ten Brummelhuis and Bakker, 2012). As a core resource in the resource caravan, it drives the operation of the entire resource system, thereby improving task quality and work efficiency (Hobfoll et al., 2018).

Similarly, self-efficacy, as a core component of individuals’ resource caravan, exerts its effect on job performance through the resource gain pathway. Enhanced by physical exercise (a resource gain), high self-efficacy enables employees to invest more effort, set challenging goals, and cope with work stressors by mobilizing other resources in their resource caravan, thus sustaining motivation and maintaining high performance quality (Chen et al., 2015; Stajkovic et al., 2018).

In summary, these mechanisms based on COR Theory explain why physical exercise influences job performance primarily through its indirect effects on positive affect and self-efficacy. Both variables, as key resources in the resource caravan, are enhanced by physical exercise and further promote the accumulation and integration of other work-related resources, ultimately improving job performance—providing theoretical support for the verification of the hypotheses.

### 4.4 Model presentation

**Figure 1:**
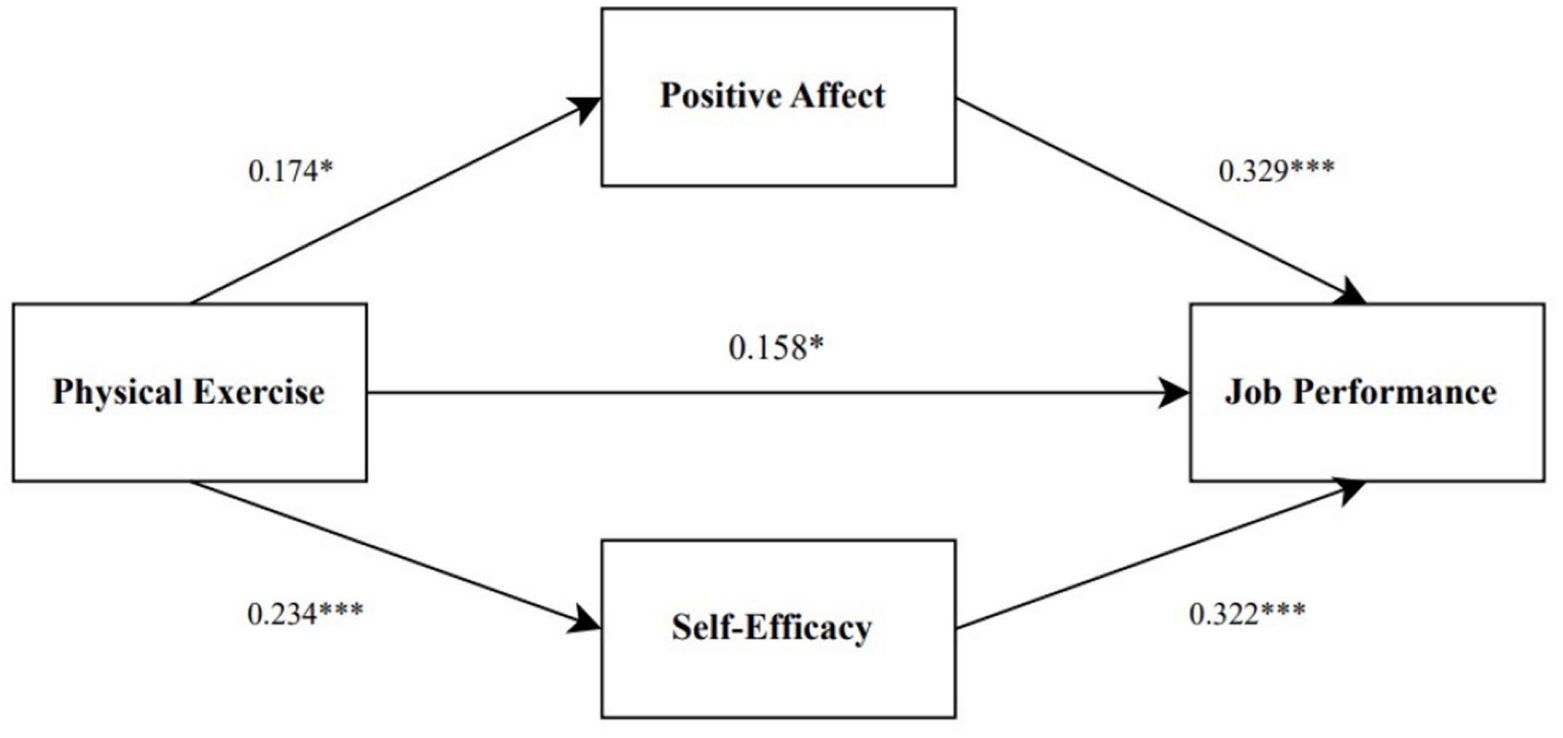
Theoretical model of the relationship between physical exercise and job performance. *p < 0.05, **p < 0.01, ***p < 0.001.

The hypothesized mediation model and standardized path coefficients are presented in Figure 1.

## 5 Discussion

This study explored the relationship between physical exercise and job performance, and examined the mediating roles of positive affect and self-efficacy. The results showed that physical exercise was significantly positively correlated with both positive affect and self-efficacy, and both variables significantly predicted job performance. Notably, when positive affect and self-efficacy were simultaneously included in the model, the direct association between physical exercise and job performance was reduced and became non-significant, highlighting the dominant role of the indirect pathways through psychological mediators. Specifically, these results indicate that the effects of physical exercise on job performance are primarily transmitted through mediating variables rather than direct pathways. As a resource gain behavior in the non-work domain, the core value of physical exercise lies in stimulating the generation and accumulation of psychological resources such as positive affect and self-efficacy, which are then transferred to the work context and transformed into improved job performance, constituting the core theoretical contribution of this study. These findings extend previous research (Fritz and Sonnentag, 2006) by clarifying the complete “physical exercise— psychological resources —job performance” indirect pathway and further enriching the application of Conservation of Resources (COR) Theory in the association between workplace outcomes and non-work behaviors.

Based on COR Theory, this study further elaborates on the theoretical implications of the mediated effect: as a typical resource gain behavior, physical exercise can effectively promote the generation, accumulation, and activation of positive affect and self-efficacy. When transferred to the work context, these psychological resources ultimately enhance job performance by improving employees’ work engagement, coping capabilities, and motivation levels—this is highly consistent with the core tenets of COR Theory, which emphasizes that individuals achieve resource appreciation and alleviate resource depletion through resource gain cycles (Hobfoll, 1989; Xanthopoulou et al., 2007). More importantly, the verification of the indirect effect clarifies the critical role of psychological resources in linking physical exercise to job performance, rather than suggesting a direct effect of exercise itself.

Positive affect and self-efficacy can be regarded as core complementary elements within the “resource caravan,” jointly supporting the full mediation mechanism: positive affect functions as an affective resource that supports coping, reduces work-related strain, and sustains effective functioning under job demands; self-efficacy, in turn, helps employees maintain positive affect under pressure, motivates them to set higher goals, and encourages persistence in overcoming work difficulties (Halbesleben et al., 2014; Hobfoll et al., 2018). Together, they form a resource gain cycle that synergistically enhances job performance. Previous meta-analytic evidence (Chang et al., 2012) has also shown that physical activity interventions can improve cognitive function and executive control, further confirming the rationality of this mediation mechanism.

Theoretically, job performance is a complex outcome shaped by the interaction of individual resources, task characteristics, and organizational demands, and its improvement relies on the coordination of multiple pathways rather than the direct impact of a single behavior. As a non-work behavior, physical exercise cannot directly alter employees’ job-specific skills, task strategies, or work processes—which may explain its non-significant direct effect. Instead, its value lies in accumulating transferable psychological resources that provide internal support for employees to cope with work challenges (Hobfoll, 1989; Xanthopoulou et al., 2007). This study further explores the potential moderating logic underlying the non-significant direct effect, addressing a gap in previous research: the direct association between physical exercise and job performance may be moderated by boundary conditions such as job type (physical exercise may have a direct effect by improving physical fitness in physically demanding jobs, while cognitive jobs rely more on the transmission of psychological resources), exercise characteristics, and individual differences—consistent with previous research conclusions (Fritz and Sonnentag, 2006; Park and Jang, 2019).

The emphasis on the mediated mechanism provides refined and actionable practical implications for organizational health promotion and performance management, moving beyond the generalized recommendation of “encouraging physical exercise.” Based on the core conclusion that “physical exercise affects job performance through psychological resources,” Organizations should strategically select exercise programs that are most effective in stimulating the mediating psychological resources identified in this study, including positive affect and self-efficacy. Specifically, evidence suggests that different exercise formats and modalities contribute differently to psychological resources: social, collaborative formats such as team sports or group classes can enhance positive affect by fostering social support and connectedness (Eather et al., 2023); aerobic exercises, particularly moderate-to-vigorous activities like running, cycling, or swimming, are especially effective in improving mood, reducing stress, and strengthening positive affect (Ensari et al., 2015); resistance or strength training can uniquely enhance self-efficacy by providing mastery and competence experiences, improving individuals’ confidence in their abilities (O’Connor et al., 2010).

Based on these findings, we recommend three types of targeted intervention strategies:

Design personalized exercise programs tailored to the psychological resource goals of different employee groups. For employees or teams needing enhanced positive affect and stress reduction, incorporate aerobic and collaborative group activities. For employees whose roles require high confidence and self-efficacy, integrate resistance or strength training exercises, or combination programs that provide mastery experiences. Subsidies, reserved exercise time, and flexible scheduling can lower participation barriers (Conn et al., 2009).

Implement systematic tracking and evaluation mechanisms. Regularly monitor employees’ engagement with different exercise programs and assess changes in psychological resources (e.g., mood, self-efficacy) and work performance. Use these data to dynamically adjust intervention plans to optimize effectiveness and ensure alignment with organizational performance goals.

Cultivate a supportive organizational climate. Leadership demonstration, team-based challenges, and incentive mechanisms can encourage participation in high-value exercise programs. Activities such as group yoga sessions, mindfulness exercises, and team sports projects can leverage social interaction and goal achievement to maximize the transfer of psychological resources to work performance (Hobfoll et al., 2018; Schaller et al., 2024).

Overall, by explicitly linking exercise type and modality to specific psychological mediators, organizations can design interventions that are both operable and theoretically grounded. This approach not only promotes employee health but also strategically enhances job performance, achieving a dual benefit for employees and the organization.

Finally, it should be noted that the reduced direct effect between physical exercise and job performance in this study does not mean that there is no universal direct association between the two; instead, it is moderated by various boundary conditions—an issue not explored in depth in the present study. Combined with the logic of existing research and the conclusions of this study, potential moderating variables mainly involve three dimensions: work context, individual differences, and psychological states. The pathway through which physical exercise affects job performance may be heterogeneous under different conditions. This finding further improves the elaboration of the mediated mechanism in this study and echoes the core viewpoint of COR Theory that “the role of resources depends on the context.”

### 5.1 Limitations

Despite its contributions, this study has several limitations that should be acknowledged. First, although a three-wave time-lagged design was employed to reduce common method bias and improve temporal separation among variables, all measures were based on self-reported questionnaires collected within a relatively short time frame. As a result, causal inferences should be drawn with caution.

In particular, while the time-lagged design strengthens temporal ordering, it does not fully capture the long-term accumulation and deployment of psychological resources implied by the proposed mediation model. Future studies would benefit from longitudinal designs with longer intervals or experimental approaches to more rigorously test the causal ordering implied in the mediation model.

Second, the exclusive reliance on self-reported performance may introduce potential biases, such as leniency, social desirability, or common method variance. However, a substantial body of meta-analytic and review evidence supports the validity of self-reported job performance, especially when the focus is on psychological predictors such as positive affect and self-efficacy. For example, Heidemeier and Moser (2009) found that self-ratings correlate moderately with supervisor ratings (*ρ* ≈ 0.34), indicating that self-assessments capture meaningful variance in actual performance. Latham and Pinder (2005) further highlighted that self-evaluations are particularly informative when examining motivational and cognitive antecedents of performance, reflecting employees’ perceptions of their own effectiveness. In addition, Conway and Lance (2010) emphasized that self-reports remain a valid and widely used method in organizational research, particularly for capturing intra-individual variations that objective or supervisory ratings may not fully reflect. Nevertheless, future research is encouraged to incorporate multi-source performance data (e.g., supervisor or peer ratings) to further enhance measurement robustness and external validity.

Third, limitations in sample representativeness should be noted. A convenience sampling approach was adopted due to practical constraints, including time, budget, and accessibility of employees across multiple regions. While convenience sampling is a widely used non-probability technique in organizational research for examining theoretically hypothesized relationships (Memon et al., 2020; Shen and Wu, 2022), it may introduce selection bias and limit statistical generalizability. Nevertheless, when grounded in robust theoretical frameworks and supported by empirical controls, convenience samples can still provide meaningful insights into associations among variables. Future research should aim to enhance representativeness by expanding geographic coverage, including participants from diverse industries, and employing stratified or quota sampling to validate and extend these findings.

Finally, although positive affect and self-efficacy fully accounted for the association between physical exercise and job performance in this study, this does not preclude the involvement of other psychological or contextual mechanisms. Variables such as work engagement, recovery experience, or broader psychological capital were not included and warrant further investigation.

### 5.2 Future directions

Future research may extend the present study in several directions. First, longitudinal or experimental studies are needed to examine the dynamic processes through which sustained physical exercise leads to long-term psychological resource accumulation, stabilization, and transfer into work-related functioning. Such designs would allow researchers to distinguish short-term affective fluctuations from more enduring resource gain cycles.

Second, future studies are strongly recommended to employ multi-source data when measuring job performance, so as to alleviate the limitations of relying solely on self-reported data and provide more robust empirical support for the proposed mediation pathways. For instance, combining self-reported psychological resources with supervisor-rated or objective performance indicators would enable a more rigorous and credible test of the resource transfer mechanism in this study.

Third, future research should broaden the sampling scope to include employees from diverse cultural, industrial, and organizational contexts. Comparative studies across job types—such as physically demanding versus cognitively demanding roles—may help clarify whether the indirect effects of physical exercise vary as a function of task characteristics and work demands.

Finally, future studies may expand the current model by incorporating additional mediators or moderators, such as job demands, organizational support for health behaviors, recovery experiences, or psychological capital. Examining these boundary conditions would contribute to a more comprehensive theoretical framework and clarify when, how, and for whom physical exercise exerts the strongest beneficial impact on job performance.

## 6 Conclusion

This study provides empirical evidence that the link between physical exercise and job performance operates primarily indirectly, mediated by employees’ psychological resources. Positive affect and self-efficacy play a dominant mediating role in this relationship, highlighting that exercise enhances work performance by fostering favorable emotional states and strengthening individuals’ confidence in their abilities. Rather than exerting a direct effect on task outcomes, physical exercise functions as an upstream resource-generating activity, enhancing internal psychological states that employees subsequently deploy in the workplace.

These findings underscore the strategic value of physical exercise beyond health benefits, positioning it as a resource-building practice that organizations can leverage to improve job performance. Encouraging regular exercise may serve as a practical organizational intervention by cultivating employees’ emotional and cognitive resources, thereby supporting sustained and adaptive work behaviors. Future research could further examine additional mediating pathways and boundary conditions to clarify how exercise-induced resources exert their indirect influence on performance across diverse work contexts.

## Data Availability

The original contributions presented in the study are included in the article/supplementary material, further inquiries can be directed to the corresponding author.
